# FOXA1 promotes tumor cell proliferation through AR involving the Notch pathway in endometrial cancer

**DOI:** 10.1186/1471-2407-14-78

**Published:** 2014-02-11

**Authors:** Meiting Qiu, Wei Bao, Jingyun Wang, Tingting Yang, Xiaoying He, Yun Liao, Xiaoping Wan

**Affiliations:** 1 Department of Obstetrics and Gynecology, International Peace Maternity & Child Health Hospital, Shanghai Jiao Tong University School of Medicine, Hengshan Road, Shanghai, China; 2 Department of Obstetrics and Gynecology, Shanghai First People’s Hospital, Shanghai Jiao Tong University School of Medicine, Xinsongjiang Road, Shanghai, China

**Keywords:** Endometrial cancer, FOXA1, AR, Proliferation, Notch pathway

## Abstract

**Background:**

Increasing evidence suggests that forkhead box A1 (FOXA1) is frequently dysregulated in many types of human cancers. However, the exact function and mechanism of FOXA1 in human endometrial cancer (EC) remains unclear.

**Methods:**

FOXA1 expression, androgen receptor (AR) expression, and the relationships of these two markers with clinicopathological factors were determined by immunohistochemistry analysis. FOXA1 and AR were up-regulated by transient transfection with plasmids, and were down-regulated by transfection with siRNA or short hairpin RNA (shRNA). The effects of FOXA1 depletion and FOXA1 overexpression on AR-mediated transcription as well as Notch pathway and their impact on EC cell proliferation were examined by qRT-PCR, western blotting, co-immunoprecipitation, ChIP-PCR, MTT, colony-formation, and xenograft tumor–formation assays.

**Results:**

We found that the expression of FOXA1 and AR in ECs was significantly higher than that in a typical hyperplasia and normal tissues. FOXA1 expression was significantly correlated with AR expression in clinical tissues. High FOXA1 levels positively correlated with pathological grade and depth of myometrial invasion in EC. High AR levels also positively correlated with pathological grade in EC. Moreover, the expression of XBP1, MYC, ZBTB16, and UHRF1, which are downstream targets of AR, was promoted by FOXA1 up-regulation or inhibited by FOXA1 down-regulation. Co-immunoprecipitation showed that FOXA1 interacted with AR in EC cells. ChIP-PCR assays showed that FOXA1 and AR could directly bind to the promoter and enhancer regions upstream of MYC. Mechanistic investigation revealed that over-expression of Notch1 and Hes1 proteins by FOXA1 could be reversed by AR depletion. In addition, we showed that down-regulation of AR attenuated FOXA1-up-regulated cell proliferation. However, AR didn’t influence the promotion effect of FOXA1 on cell migration and invasion. In vivo xenograft model, FOXA1 knockdown reduced the rate of tumor growth.

**Conclusions:**

These results suggest that FOXA1 promotes cell proliferation by AR and activates Notch pathway. It indicated that FOXA1 and AR may serve as potential gene therapy in EC.

## Background

Endometrial cancer (EC) is one of the most common gynecologic malignancies. The incidence of EC has markedly increased in recent years. EC is broadly classified into two groups [1]; type I ECs are linked to estrogen excess, hormone-receptor positivity, and favorable prognoses, whereas type II, primarily serous tumors, are more common in older women and have poorer outcomes [2]. Primary treatment, including surgery and radiation, cannot provide sufficient tumor control, especially in high-grade, undifferentiated tumors with deep muscle infiltration. Endocrine treatment, including medroxyprogesterone acetate or tamoxifen, is sometimes useful to improve the outcome. However, patients with type II EC and even some patients with type I EC are refractory to traditional endocrine treatment [3]. Thus, a new treatment is needed to achieve a better response.

Several studies have shown that the majority of ECs also express another hormone receptor, androgen receptor (AR) [4,5]. The results of immunohistochemical analysis indicate that, compared with endometrial glandular epithelial cells in normal cycling endometrium, more epithelial cells express AR in ECs [4]. Moreover, in female mice, in contrast to AR−/− uteri, AR+/+ uteri have uterine hypertrophy and endometrial growth [6]. It thus is very important to examine the possible actions and metabolism mediated by AR in human EC.

Forkhead box A1 (FOXA1) is a transcription factor that belongs to the forkhead family consisting of the winged-helix DNA-binding domain and the N-terminal and C-terminal transcriptional domains. FOXA1 is expressed in various organs, including breast, liver, pancreas, and prostate, and can influence the expression of a large number of genes associated with metabolic processes, regulation of signaling, and the cell cycle [7,8]. FOXA1 has been identified as a “pioneer factor” that binds to chromatin-packaged DNA and opens the chromatin for binding of additional transcription factors, including AR [9]. FOXA1 also binds directly to AR and regulates transcription of prostate-specific genes in prostate cancer [10]. Recent global gene expression studies of prostate cancer and triple-negative breast cancer have shown that high FOXA1 expression, which correlates positively with AR level, promotes tumor proliferation [11,12]. Thus, FOXA1 expression is considered a predictor of poor survival in prostate cancer and triple-negative breast cancer. However, the interaction between FOXA1 and AR in EC remains unclear.

An aberrant Notch pathway has been documented in various cancer types and has been associated with tumorigenesis [13-15]. The Notch pathway is initiated by ligand binding, which is followed by intramembranous proteolytic cleavage of the Notch1 receptor to release an active form of the Notch intracellular domain (NICD). The NICD subsequently translocates to the nucleus and acts as a transcriptional activator to enhance the expression of target genes such as Hairy-enhancer of split1 (Hes1) [16]. Abnormal activation of the Notch pathway promotes proliferation in a variety of cancer cell types, including EC [15,17].

In the present study, we investigated the dependency of AR on FOXA1 expression in tissue paraffin sections, in multiple cellular contexts, and on tumor-bearing nude mice. Here we show, for the first time, that FOXA1 activates the Notch pathway through AR and that AR is required for FOXA1-enhanced cell proliferation in EC.

## Methods

### Patients and tissues

A total of 57 normal endometrial samples, 11 atypical hyperplasias, and 76 EC specimens obtained from Chinese female patients who underwent surgical treatment from 2011 to 2013 at the Shanghai Jiao Tong University Affiliated International Peace Maternity & Child Health Hospital (Shanghai, China) were available for examination in this study. Tissues were embedded in paraffin. Two independent pathologists verified the histological diagnosis of all collected tissues. No patient had received neoadjuvant therapy or endocrine therapy before the surgery. The clinicopathological characteristics of EC patients are presented in Table 1. The samples of EC, atypical hyperplasias and normal endometrial tissues were collected after written informed consent from the patients. The Human Investigation Ethical Committee of the International Peace Maternity & Child Health Hospital Affiliated Shanghai Jiao Tong University approved this study.

**Table 1:** The relationship between protein expression and clinicopathological features in EC

**Parameter**	**n**	**FOXA1**		**p**	**AR**		**p**
		**High**	**Low**		**High**	**Low**	
Age							
≤55	31	21	10	0.916	19	12	0.493
>55	45	31	14		31	14	
FIGO stage							
I–II	64	41	23	0.059	41	23	0.464
III–IV	12	11	2		9	3	
Pathological type							
Adenocarcinoma	63	47	16	0.469	41	22	0.774
Papillary serous carcinoma	13	10	3		9	4	
Histological grade							
G1	35	19	16	0.038	18	17	0.040
G2	22	17	5		18	4	
G3	6	6	0		5	1	
Lymph node metastasis							
Positive	7	6	1	0.271	5	2	0.86
Negative	66	43	23		45	21	
Depth of myometrial invasion							
≤1/2	47	28	19	0.035	29	18	0.339
>1/2	29	24	5		21	8	
ER_α_ expression							
Positive	61	43	18	0.434	41	20	0.598
Negative	15	9	6		9	6	
P53 expression							
Positive	20	14	6	0.86	15	5	0.312
Negative	56	38	18		35	21	

### Immunohistochemical staining

Staining was performed on paraffin-embedded specimens using primary antibodies as follows: anti-FOXA1 (1:200; Abcam, Cambridge, MA, USA) and anti-AR (1:50; Abcam). The percentage of positively stained cells was rated as follows: 0 point = 0%, 1 point = 1% to 25%, 2 points = 26% to 50%, 3 points = 51% to 75%, and 4 points = greater than 75%. The staining intensity was rated in the following manner: 0 points = negative staining, 1 point = weak intensity, 2 points = moderate intensity, and 3 points = strong intensity. Then, immunoreactivity scores for each case were obtained by multiplying the values of the two parameters described above. The average score for all of five random fields at 200× magnification was used as the histological score (HS). Tumors were categorized into two groups based on the HS: low-expression group (HS = 0–5) and high-expression group (HS = 6–12).

### Cell culture and experimental setup

The human endometrial cell lines AN3CA, RL95-2, and HEC-1B were obtained from the Chinese Academy of Sciences Committee Type Culture Collection cell bank. These three cell lines were grown in Dulbecco’s modified Eagle’s medium (DMEM)/F12 (HyClone, Waltham, MA, USA) supplemented with 10% fetal bovine serum (Gibco, Carlsbad, CA, USA) in a humidified atmosphere of 5% CO_2_ at 37°C. The human endometrial cell line MFE-296 was purchased from Sigma (St. Louis, MO, USA). The MFE-296 cell line was grown in high-glucose DMEM (4.5 g/L glucose) (HyClone) supplemented with 10% fetal bovine serum in a humidified atmosphere of 5% CO_2_ at 37°C.

To investigate the impact of FOXA1 on the AR-mediated transcription, the AR pathway agonist 5α-dihydrotestosterone (DHT) (Dr. Ehrenstorfer, Augsburg, Germany) and the AR pathway blocker flutamide (Sigma) were purchased and dissolved in 100% ethanol for storage. In this study they were diluted with phenol red–free DMEM/F12 (Gibco) immediately before each experiment, with the final concentration of ethanol at 0.1%. DHT was added into the cell culture media at concentrations of 10^−9^ to 10^−7^ M for different periods (0–48 h). To block the activation of AR-mediated transcription, flutamide (10^−6^ M) was added into the media 30 min before DHT. Vehicle contained 0.1% absolute ethanol/phenol red–free DMEM/F12.

### Stable transfection

To stably knock down endogenous FOXA1 expression, MFE-296 cells were grown to 30% confluency in 6-well culture plates and then infected with lentivirus carrying an shRNA targeting FOXA1 (shFOXA1) or a negative control vector (NC; LV3-pGLV-h1-GFP-puro vector, D03004; GenePharma, Shanghai, China) at a multiplicity of infection of 70 in the presence of polybrene (8 μg/mL). After 48 h of infection at 37°C, the medium was replaced with fresh medium and incubated further for 72 h before analysis using quantitative RT-PCR (qRT-PCR) and western blotting for FOXA1 expression. The shRNA sequences used were 5′-GAGAGAAAAAAUCAACAGC-3′ (shFOXA1) and 5′-TTCTCCGAACGTGTCACGT-3′ (NC).

### Transient transfection

The plasmid PWP1/GFP/Neo-AR containing transfection-ready AR cDNA (exAR) and its negative control PWP1/GFP/Neo were gifts from Doctor Yuyang Zhao at Shanghai First People’s Hospital. MFE-296 cells stably transfected with shFOXA1 or NC were transiently cotransfected with PWP1/GFP/Neo-AR (exAR) or its negative control (NC). The plasmid pCMV/3FLAG/Neo-FOXA1 containing transfection-ready FOXA1 cDNA (exFOXA1) (GenBank: BC033890) and a pure pCMV/3FLAG/Neo (NC) were purchased from Genechem (Product code: GOSE33403; Shanghai, China). AN3CA cells were transiently transfected with exFOXA1 or NC or cotransfected with a siRNA targeting AR (siAR) (Genephama Biotech, Shanghai, China) or its negative control (NC) in Opti-MEM (Invitrogen, Carlsbad, CA, USA) using Lipo2000 (Invitrogen). The siRNA targeting FOXA1 (siFOXA1) and its negative control (NC) were purchased from Genephama Biotech (Shanghai, China). AN3CA cells were transiently transfected with exAR or NC or cotransfected with siFOXA1 or NC in Opti-MEM (Invitrogen) using Lipo2000 (Invitrogen). The transfection solution was removed from the cells and replaced with standard medium after 8 h. The sequences of the siRNA oligos used were: siAR: sense: 5′-AUGUCAACUCCAGGAUGCUTT-3′, antisense: 5′-AGCAUCCUGGAGUUGACAUTT-3′; siFOXA1: sense: 5′-GAGAGAAAAAAUCAACAGC-3′, antisense: 5′-GCUGUUGAUUUUUUCUCUC-3′.

### qRT-PCR

Total RNA was extracted from cultured cells by Trizol Reagent (Invitrogen). RNA was converted to cDNA with the one-step Prime Script RT reagent kit (TaKaRa, Dalian, China), and the cDNA was analyzed by real-time PCR using SYBR Premix Ex Taq (TaKaRa) in an Eppendorf Mastercycler® realplex. Each sample was assayed in triplicate in each of three independent experiments. All values are expressed as mean ± standard deviation. The following primers were used: FOXA1: sense: 5′-AGGTGTGTATTCCAGACCCG-3′, antisense: 5′-TTGACGGTTTGGTTTGTGTG-3′; AR: sense: 5′-CCTGGCTTCCGCAACTTACAC-3′, antisense: 3′-GGACTTGTGCATGCGGTACTCA-5′; MYC: sense: 5′-AAAGGCCCCCAAGGTAGTTA-3′, antisense: 5′-TTTCCGCAACAAGTCCTCTT-3′; XBP1: sense: 5′-CCTTGTAGTTGAGAACCAGG-3′, antisense: 5′-GGGGCTTGGTATATATGTGG-3′; UHRF1: sense: 5′-AAGGTGGAGCCCTACAGTCTC-3′, antisense: 5′-CACTTTACTCAGGAACAACTGGAAC-3′; and ZBTB16: sense: 5′-CCAGCAGATTCTGGAGTATGCA-3′, antisense: 5′-GCATACAGCAGGTCATCCAAGTC-3′.

### Western blotting

Total protein was extracted using a RIPA kit (Beyotime, Shanghai, China) containing a 1% dilution of the protease inhibitor PMSF (Beyotime). Protein concentrations were determined by the enhanced BCA Protein Assay kit (Beyotime). Equal amounts of protein in each lane were separated by 8% SDS-PAGE and transferred to a PVDF membrane (Millipore, Billerica, MA, USA). After blocking the membrane in blocking buffer (5% milk powder in 20 mM Tris–HCl pH 7.5, 500 mM NaCl, 0.1% (v/v) Tween 20), the membrane was incubated with primary antibodies against FOXA1 (1:1000; Abcam), AR (1:2000; Cell Signaling Technology, Danvers, MA, USA), Notch1 (1:2000; Epitomics, Burlingame, CA, USA), Hes1 (1:2000; Epitomics), and β-actin (1:2000, Cell Signaling Technology) at 4°C overnight. Peroxidase-linked secondary anti-rabbit or anti-mouse antibodies were used to detect the bound primary antibodies.

### Co-immunoprecipitation

Total protein was extracted from cells treated or not treated with 10^−7^ M DHT for 24 h (described in the Cell culture and experimental setup section). After protein quantification, 500 μg of each cell lysate was added to 10 μl of anti-FOXA1 (Epitomics) and shaken at 4°C overnight, then added to 30 μl of Protein A + G Agarose (Beyotime), shaken at 4°C for 4 h, centrifuged at 2500 × *g* for 5 min, and washed with a RIPA kit (Beyotime) to collect the immunoprecipitate-bound agarose beads. Each immunoprecipitate was denatured with 20 μl of 1× SDS-PAGE loading buffer at 100°C for 5 min. Each supernatant was subjected to SDS-PAGE (8% acrylamide). It is important to note that FOXA1 (51 kDa) is close in size to IgG (55 kDa). To avoid detecting IgG protein left from the immunoprecipitation process and FOXA1 protein from the same species in the western blot at the same time, we used anti-FOXA1 from mouse in western blotting, whereas anti-FOXA1 from rabbit was used for immunoprecipitation. Primary antibodies against AR (1:2000) and FOXA1 (1:500; Santa Cruz Biotechnology, Dallas, TX, USA) were used for western blotting. Other steps were as described in the Western blotting section.

### Chromatin immunoprecipitation (ChIP)-PCR

Chromatin immunoprecipitation (ChIP) assays were performed as previously described [18] using anti-FOXA1 antibody (ab23738, Abcam), anti-AR antibody (sc-7305, Santa Cruz Biotechnology). FOXA1-AR overlapping binding sites were identified by Chip-seq as previously depicted [19] and by qRT-PCR using SYBR Premix Ex Taq (Takara). Enrichment was calculated using the comparative Ct method, and was analyzed for specificity, linearity range, and efficiency in order to accurately evaluate the occupancy (percentage of immunoprecipitation/input). IgG was used as negative control. The primers used included: MYC pro: sense: 5′-CCCCCGAATTGTTTTCTCTT-3′, antisense: 5′-TCTCATCCTTGGTCCCTCAC-3′; MYC enh-1: sense: 5′-AGACAGAGGCAGGGTGGAG-3′, antisense: 3′-CCCAGGTAAACAGCCAATGT-5′; MYC enh-2a: sense: 5′-CCGTTCCGTGTCTAACCACT-3′, antisense: 5′-ATGAAACTCGGGGAGTGTTG-3′; MYC enh-2b: sense: 5′-AGCGTTCTCTTTGCCAGAAA-3′, antisense: 3′-GGCAAAGCTTCACAGAGGAC-5′; MYC enh-2c: sense: 5′-CACACAAGAAGAGCAAACTGAAG-3′, antisense: 5′-TGAGGATTGTTAGGAATCTCTGG-3′.

### MTT assay

Cells (3 × 10^3^ cells/well) were plated in 96-well plates. Then, 20 μl of 3-(4,5-dimethylthiazol-2-yl)-2,5-diphenyltetrazolium bromide (MTT, 5 mg/ml; Sigma) was added to each well and subsequently incubated at 37°C for 4 h. The absorbance at 490 nm was then measured using a microplate reader. Cells incubated with culture medium were used as a control group. Each sample was assayed in triplicate.

### Colony-formation assays

Cell lines were trypsinized to generate a single-cell suspension, and 120 cells/well (MFE-296 cells) or 200 cells/well (AN3CA cells) were seeded into 6-well plates. Dishes were returned to the incubator for 14 days, and the colonies were fixed with methanol for 30 min at room temperature and then stained with 0.5% crystal violet for 1 h.

### Cell migration and invasion assays

Cells were trypsinized, centrifuged, and resuspended in serum-free medium. Cells were then plated at a density of 1 × 10^5^/well (for the migration assay) or 2 × 10^5^/well (for the invasion assay) in invasion chambers (8 μm pore size; BD Biosciences, California, USA) with or without matrigel coating for invasion and migration assays. Complete medium (600 μl) was added to the lower chamber as a chemoattractant. After incubation for 5 h (MFE-296) or 24 h (AN3CA) for the migration assay, or after incubation for 24 h (MFE-296) or 48 h (AN3CA) for the invasion assay, cells were fixed with 4% paraformaldehyde for 1 h. Cells on the apical side of each insert were removed by mechanical scraping. Cells that migrated to the basal side of the membrane were stained with 0.5% crystal violet and counted at 200× magnification. The migration and invasion assays were repeated at least three times.

### Xenograft tumor–formation assays

Female athymic mice of 4 weeks of age were obtained from the Shanghai Experimental Animal Center of the Chinese Academy of Science. Our animal research was carried out in strict accordance with the recommendations in the Guideline for the Care and Use of Laboratory Animals of China. The protocol was approved by the Committee on the Ethics of Animal Experiments of the Obstetrical and Gynecological Hospital affiliated Fudan University (Permit Number: SYXK (hu) 2008–0064). All efforts were made to minimize animal suffering.

To establish a nude mouse model bearing EC, uninfected MFE-296 cells (MFE-296), stable MFE-296 cells infected with lentivirus carrying shFOXA1 (MFE-296/shFOXA1) or vector alone (MFE-296/NC) were used. All mice were randomly divided into three groups of four mice. Each mouse was given a unilateral subcutaneous injection of 1 × 10^7^ cells. Tumor measurement began one week after injection and was conducted weekly using digital calipers. The tumors were removed and weighed after 42 days. Tumor volume was calculated as follows: tumor volume (cm^3^) = (the longest diameter) × (the shortest diameter)^2^ × 0.5.

### Immunohistochemical staining of mouse tumor samples

Tumor samples from xenografted mice were collected and fixed according to routine procedures. Histological staining was then performed on the tissue sections of the paraffin-embedded tumors using the streptavidin-biotin-peroxidase method. Primary antibodies were as follows: anti-FOXA1 (1:200; Abcam), anti-AR (1:50, Abcam), anti-Notch1 (1:100; Epitomics,), anti-Hes1 (1:250; Epitomics), anti-Ki67 (1:100; Boster, Wuhan, China), and anti-PCNA (1:100; Boster). The sections were then counterstained with hematoxylin and eosin (H&E).

### Statistics

Measured data were assessed by unpaired Student’s *t*-test or one-way ANOVA for multiple comparisons, and χ2 test for 2 × 2 tables was used to compare the categorical data. p < 0.05 was considered significant.

## Results

### Expression of FOXA1 and AR in endometrial tissues and the clinicopathological significance in EC specimens

**Figure 1:**
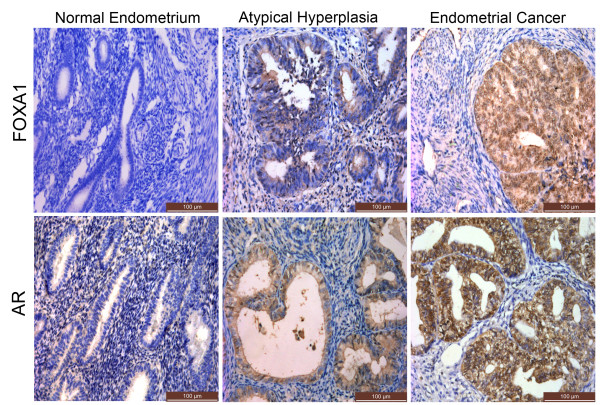
**Immunohistochemical staining of FOXA1 and AR in normal endometrium, atypical hyperplasias, and endometrial cancer.** FOXA1 and AR expression in normal endometrium, atypical hyperplasias and endometrial cancer. (Immunohistochemical staining, ×200).

We assessed relative FOXA1 and AR levels in EC samples, atypical hyperplasias, and normal endometrial tissue samples using immunohistochemistry. FOXA1 was higher in atypical hyperplasias and even higher in EC compared with normal endometrial tissues (p = 0.005) (Figure 1, Additional file 1: Table S1). Notably, the expression of AR was also significantly higher in EC (p = 0.033) (Figure 1, Additional file 2: Table S2). The results also showed that FOXA1 expression correlated positively with AR expression (p = 0.003) (Table 2). Correlation analysis between FOXA1 and pathological grade of EC showed that FOXA1 expression was higher in G3 tumors (6/6) compared with either G2 (17/22) or G1 (19/35) tumors (p = 0.038) (Table 1). Significantly higher FOXA1 expression was also found in tumors that displayed a greater depth of myometrial invasion (p = 0.035). Finally, our results also indicated that AR was much higher in G3 and G2 tumors compared to G1 (p = 0.040) (Table 1). These results suggested that FOXA1 expression, which correlated with AR expression, had a connection with the development of EC and risk-associated clinical features of the disease.

**Table 2:** Immunohistochemical analysis of protein expression in different endometrial tissues

	**Normal endometrium**		**Atypical hyperplasia**		**Endometrial cancer**		*p
	**Negative**	**Positive**	**Negative**	**Positive**	**Negative**	**Positive**	
FOXA1	32	25	4	7	24	52	
n	57		11		76		
AR	30	27	5	6	26	50	0.003
n	57		11		76		

*The relationship between FOXA1 and AR expression was assessed by χ^2^ test.

### FOXA1 affects AR expression in human EC cells

**Figure 2:**
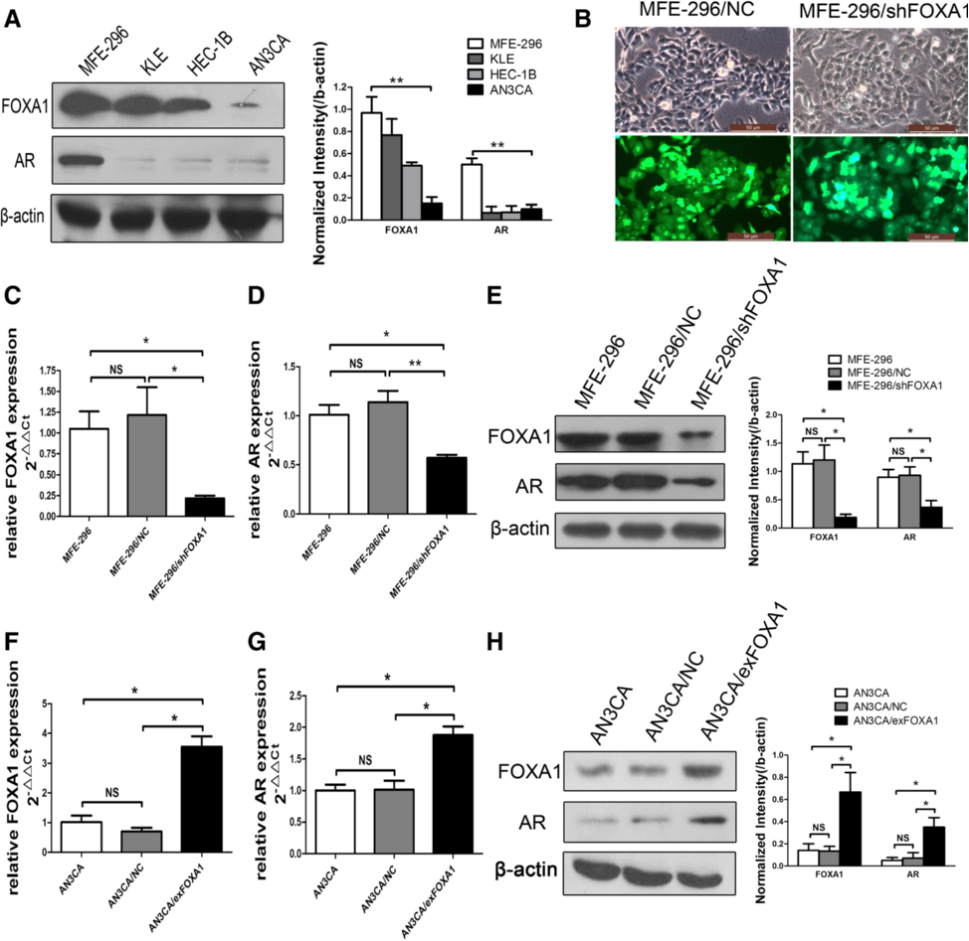
**FOXA1 affects the expression of AR in human EC cells. A**: FOXA1 and AR expression in the indicated EC cell lines as determined were measured by western blotting (Left), and further quantified by densitometry of triplicate experiments (Right). β-actin was used as a loading control. **B**: Stable transfection of MFE-296 cells with negative control vector (MFE-296-NC) or shFOXA1 (MFE-296-shFOXA1). By comparing the cells in white light (the upper panels) with the cells in green fluorescence (the lower panels), the percentage of transfected/fluorescing cells was estimated at >85%. Magnification, ×400. **C**: Quantification of FOXA1 mRNA by qRT-PCR in untransfected MFE-296 (MFE-296), MFE-296 transfected with shRNA control plasmid (MFE-296/NC), and MFE-296 transfected with shFOXA1 (MFE-296/shFOXA1). **D**: Quantification of AR mRNA by qRT-PCR in MFE-296, MFE-296/NC, and MFE-296/shFOXA1 cells. **E**: FOXA1 and AR expression in MFE-296, MFE-296/NC and MFE-296/shFOXA1 cells were measured by western blotting (Left), and further quantified by densitometry of triplicate experiments (Right). **F**: Quantification of FOXA1 mRNA by qRT-PCR in untransfected AN3CA (AN3CA), AN3CA transfected with control plasmid (AN3CA/NC), and AN3CA transfected with FOXA1 expression plasmid (AN3CA/exFOXA1). **G**: Quantification of AR mRNA by qRT-PCR in AN3CA, AN3CA/NC, and AN3CA/exFOXA1 cells. **H**: AR and FOXA1 expression in AN3CA, AN3CA/NC and AN3CA/exFOXA1 cells were measured by western blotting (Left), and further quantified by densitometry of triplicate experiments (Right). *p < 0.05, **p < 0.01, NS p > 0.05 compared with NC.

We used western blotting to examine FOXA1 and AR expression in EC cells. FOXA1 was upregulated in MFE-296 cells compared with KLE, HEC-1B, and AN3CA cells. Furthermore, the AR level was also markedly higher in MFE-296 cells than in the other three EC cell lines (Figure 2A).

We next manipulated FOXA1 expression and examined its influence on AR expression. AN3CA cells were transiently transfected with a FOXA1 plasmid to overexpress FOXA1 (AN3CA/exFOXA1) or with control vector (AN3CA/NC). Moreover, to knock down FOXA1 expression, MFE-296 cells were stably transfected with FOXA1 shRNA (MFE-296/shFOXA1) or control vector (MFE-296/NC) (Figure 2B). AR expression was then analyzed by qRT-PCR and western blotting, which showed that the AR level was significantly enhanced by FOXA1 overexpression and reduced by FOXA1 depletion (Figure 2C–H). Together, the data suggested that FOXA1 affected the AR level in EC cells.

### FOXA1 expression affects AR target gene expression in human EC cells

**Figure 3:**
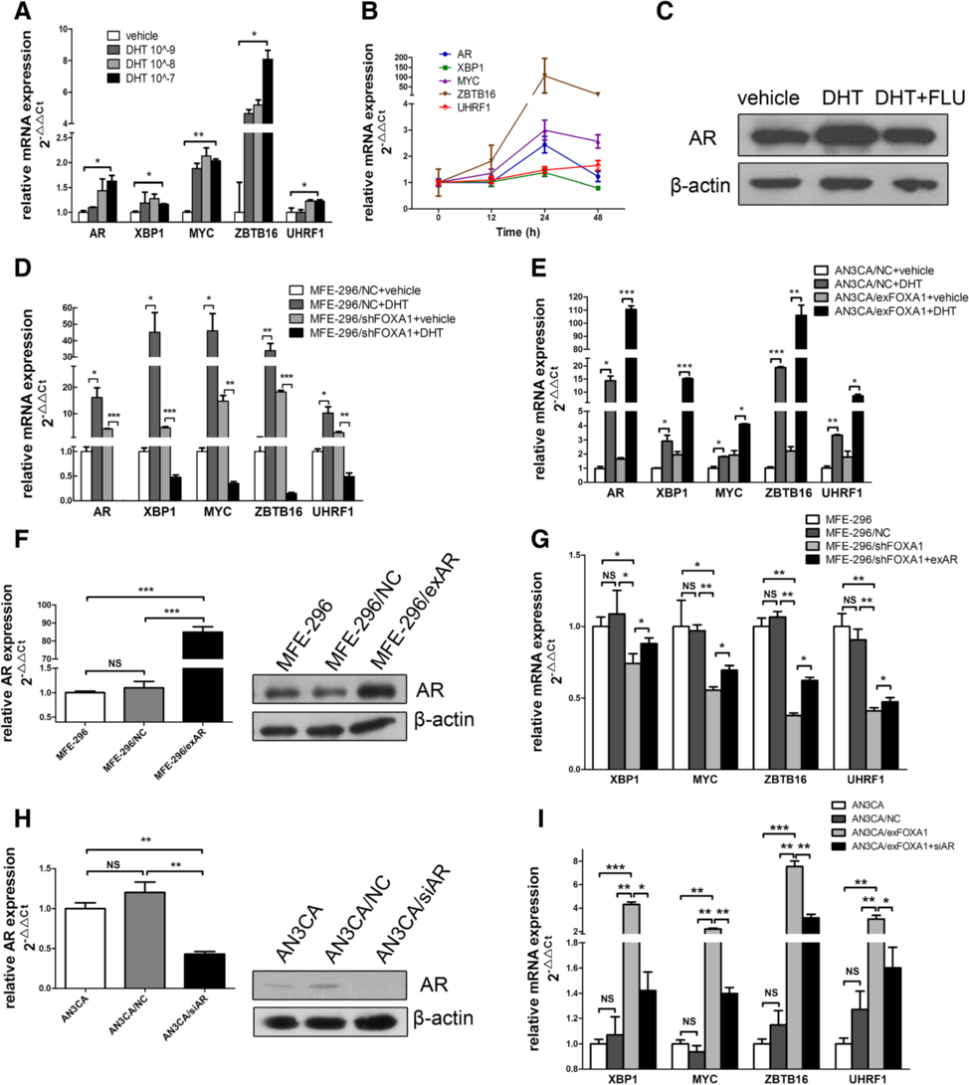
**FOXA1 affects AR-mediated transcription. A**: MFE-296 cells were treated with DHT (10^−9^ to 10^−7^ M) or vehicle (control) for 24 h. qRT-PCR was used to assess the levels of AR, XBP1, MYC, ZBTB16, and UHRF1 mRNA. The levels of each mRNA are shown relative to the level expressed in the vehicle sample. **B**: Quantification of AR, XBP1, MYC, ZBTB16, and UHRF1 mRNA by qRT-PCR in MFE-296 cells treated with 10^−7^ M DHT for 0–48 h. **C**: Western blotting analysis of AR in MFE-296 cells treated with vehicle, 10^−7^ M DHT, or 10^−7^ M DHT plus 10^−6^ M flutamide (DHT + FLU) for 24 h. β-actin was used as a loading control. **D**: MFE-296/NC and MFE-296/shFOXA1 cells were treated with 10^−7^ M DHT or vehicle for 24 h followed by qRT-PCR analysis of AR, XBP1, MYC, ZBTB16, and UHRF1 mRNA. **E**: AN3CA/NC and AN3CA/exFOXA1 cells were treated with 10^−7^ M DHT or vehicle for 24 h followed by qRT-PCR analysis of AR, XBP1, MYC, ZBTB16, and UHRF1 mRNA. **F**: Quantification of AR expression by qRT-PCR and western blotting in untransfected MFE-296 cells (MFE-296) and MFE-296 cells transfected with NC (MFE-296/NC) or exAR (MFE-296/exAR). **G**: Expression of XBP1, MYC, ZBTB16, and UHRF1 mRNA in untransfected MFE-296 cells and MFE-296 cells transfected with NC, shFOXA1, or shFOXA1 and exAR was measured by qRT-PCR. **H**: Quantification of AR expression by qRT-PCR and western blotting in untransfected AN3CA cells (AN3CA) and AN3CA cells transfected with NC (AN3CA/NC) or siAR (AN3CA/siAR). **I**: Expression of XBP1, MYC, ZBTB16, and UHRF1 mRNA in untransfected AN3CA cells and AN3CA cells transfected with NC, exFOXA1, or exFOXA1 and siAR was measured by qRT-PCR. *p < 0.05, **p < 0.01, ***p < 0.001, NS p > 0.05.

We next examined whether the FOXA1 level impacted the expression of AR target genes in EC cells. MFE-296 cells were hormone deprived and treated with vehicle or the AR pathway agonist DHT [20], and then the expression of AR target genes (XBP1, MYC, ZBTB16, and UHRF1) [12] was evaluated by qRT-PCR. This analysis confirmed that the expression of these four genes increased after treatment with DHT. Furthermore, the dose-response study (0–10^−7^ M DHT) and time-response study (0–48 h) indicated that 10^−7^ M DHT and 24 h of incubation elicited the strongest expression of AR and its target genes (Figure 3A and 3B). These data confirmed that these four genes were downstream of the AR-mediated transcription in EC cells. To partially confirm the promoting effect of DHT on AR-mediated transcription at the protein level, AR expression was examined by western blotting; DHT acted as an agonist, whereas the addition of the AR antagonist flutamide [21] reduced the DHT-enhanced expression of AR in MFE-296 cells (Figure 3C).

To investigate whether FOXA1 influences AR-mediated transcription, we transfected hormone-deprived EC cells with shFOXA1, exFOXA1, or the appropriate negative control vector and then treated them with vehicle or DHT for 24 h. In MFE-296/NC cells, DHT caused a ≥10-fold increase in the expression of the four AR-regulated genes compared with the MFE-296/NC cells treated with vehicle. When FOXA1 expression was knocked down in MFE-296 cells transfected with shFOXA1, however, the expression of these genes was not as markedly increased, and their expression decreased by 8- to 20-fold after treatment with DHT (Figure 3D). Moreover, we found that the increase in the expression of AR and AR-regulated genes was remarkably greater by DHT in the AN3CA/exFOXA1 cells compared with the AN3CA/NC cells (Figure 3E). Our findings indicated that FOXA1 expression globally affected AR-mediated transcription, with all of the four AR-regulated genes requiring FOXA1 for appropriate AR-mediated regulation.

### FOXA1 promotes AR target gene expression by interaction with AR

FOXA1 can target a series of transcription factors representing anywhere from several to hundreds of genes. To address whether the effects of FOXA1 on AR downstream targets are primarily through upregulating AR, rather than upregulating AR downstream targets directly, we used untransfected MFE-296 cells (MFE-296) and MFE-296 cells transfected with shFOXA1 (MFE-296/shFOXA1), NC (MFE-296/NC), or shFOXA1 together with exAR (MFE-296/shFOXA1 + exAR). qRT-PCR and western blotting analysis confirmed that transfection of exAR resulted in overexpression of AR (Figure 3F). qRT-PCR verified that MFE-296/shFOXA1 cells exhibited substantial decreases in the four AR targets compared with MFE-296/NC cells (Figure 3G). Furthermore, cotransfection with exAR rescued the inhibited expression of the target genes caused by FOXA1 downregulation in MFE-296/shFOXA1 cells (Figure 3G). In addition, we used untransfected AN3CA cells (AN3CA) and AN3CA cells transfected with NC (AN3CA/NC), exFOXA1 (AN3CA/exFOXA1), or exFOXA1 together with siAR (AN3CA/exFOXA1 + siAR). qRT-PCR and western blotting analysis confirmed that transfection with siAR resulted in silencing of AR (Figure 3H). Overexpression of FOXA1 increased the expression of the four AR target genes. Moreover, cotransfection with siAR partially reversed the FOXA1-induced overexpression (Figure 3I). These results verified that AR downregulation attenuated the effect of FOXA1 on AR-mediated transcription and suggested that FOXA1 might promote AR downstream targets at least in part through AR.

### FOXA1 and AR are found in the same protein complex

**Figure 4:**
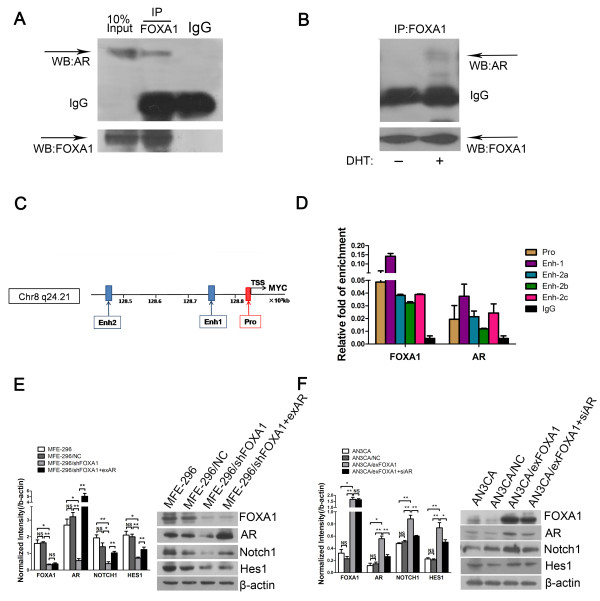
**FOXA1 affects AR-mediated transcription via binding with AR and activates the Notch pathway. A**: Co-immunoprecipitation (IP) of FOXA1 with AR in MFE-296 cells. WB: western blotting. **B**: Co-immunoprecipitation of FOXA1 with AR in AN3CA cells treated with 10^−7^ M DHT or vehicle. **C**: Schematic representation of the MYC locus. FOXA1-binding sites and AR-binding sites upstream of the TSS of MYC were predicted by ChIP-seq analysis. ChIP-PCR assays were performed using anti-FOXA1 antibody or anti-AR antibody. Pro: promoter region; Enh-1: enhancer 1 region; End-2: enhancer 2 region; TSS: transcription starting sites. **D**: Immunoprecipitated DNA fragments in ChIP-PCR assays were examined by qRT-PCR. Each sample was assayed in triplicate in each of three independent experiments. IgG was used as negative control. Primers were designed specifically for the promoter region (Pro), the enhancer 1 region (Enh-1), and the three putative FOXA1-AR binding sites within enhancer 2 region (Enh-2a, Enh-2b, and Enh-2c) according to the study [19]. **E**: Protein levels of FOXA1, AR, Notch1, and Hes1 in untransfected MFE-296 cells (MFE-296) and MFE-296 cells transfected with NC (MFE-296/NC), shFOXA1 (MFE-296/shFOXA1), or shFOXA1 and exAR (MFE-296/shFOXA1 + exAR) were measured by western blotting (Right), and further quantified by densitometry of triplicate experiments (Left). **F**: Protein levels of FOXA1, AR, Notch1, and Hes1 in untransfected AN3CA cells (AN3CA) and AN3CA cells transfected with NC (AN3CA/NC) , exFOXA1 (AN3CA/exFOXA1), or exFOXA1 and siAR (AN3CA/exFOXA1 + siAR) were measured by western blotting (Right), and further quantified by densitometry of triplicate experiments (Left). β-actin was used as a loading control. *p < 0.05, **p < 0.01 and NS p > 0.05.

To investigate whether FOXA1 affects AR-mediated transcription through binding to AR, we performed co-immunoprecipitation experiments. We used nuclear lysates from MFE-296 cells to conduct immunoprecipitation with anti-FOXA1. FOXA1 co-immunoprecipitated with AR, whereas immunoprecipitation with the isotype IgG control did not pull down AR or FOXA1 (Figure 4A), indicating that FOXA1 interacted with AR in MFE-296 cells. We also performed the co-immunoprecipitation experiment in AN3CA cells, which has low level of AR. As shown in Figure 4B, AR could be immunoprecipitated by anti-FOXA1 in the presence of DHT but not in its absence. This result indicated that FOXA1 and AR interacted physically. It is likely that FOXA1 affects AR-mediated transcription via binding with AR.

We further examined whether FOXA1 and AR could bind to the five putative FOXA1-AR binding regions, including the promoter and enhancer regions upstream of the TSS (transcription start sites) of AR target genes such as MYC (Figure 4C). Our ChIP assays showed that both FOXA1 and AR could bind to all the five putative FOXA1-AR-binding regions in MFE-296 cells. Moreover, both FOXA1 and AR bound most greatly to the Enh-1 (enhancer 1) region among the five binding regions (Figure 4D). Our ChIP data together with our co-immunoprecipitation data suggested that FOXA1 forming protein complex with AR might bind to FOXA1-AR overlapping binding regions upstream of MYC, leading to MYC activation in EC cells.

### AR is required for FOXA1-enhanced Notch pathway activation of EC cells

Pathway analysis in liver cancer shows that FOXA1/AR dual target genes are most involved in the cellular growth/proliferation pathway [22]. Notch pathway activation appears to affect proliferation in many cancers. In EC, the Notch pathway has also been shown to be involved in cell proliferation [17]. Thus, we considered that the interaction between FOXA1 and AR might be related with the Notch pathway. We used western blot analysis to assess the levels of Notch1 and the Notch pathway target protein, Hes1, in MFE-296/shFOXA1 and AN3CA/exFOXA1 cells after exAR or siAR cotransfection, respectively. Cotransfection with exAR rescued the decreased expression of Notch1 and Hes1 caused by FOXA1 downregulation in MFE-296/shFOXA1 cells (Figure 4E). Furthermore, cotransfection with siAR attenuated the increased expression of Notch1 and Hes1 caused by upregulation of FOXA1 in AN3CA/exFOXA1 cells (Figure 4F). These results suggested that the effects of FOXA1 on Notch pathway activation were mediated by AR. In order to determine whether AR was required for FOXA1-enhanced Notch pathway activation, we over-expressed AR expression in AN3CA cells, which has low level of AR. We assessed the levels of Notch1 and Hes1 in untransfected AN3CA cells (AN3CA) as well as AN3CA cells transfected with NC (AN3CA/NC), exAR (AN3CA/exAR), or exAR together with siFOXA1 (AN3CA/exAR + siFOXA1). AN3CA/exAR cells exhibited a substantial increase in AR expression as compared to AN3CA/NC cells, accompanied by over-expression of Notch1 and Hes1 (Additional file 3: Figure S1). Furthermore, cotransfection with siFOXA1 did not rescue the activation of Notch1 and Hes1 caused by AR upregulation in AN3CA/exAR cells (Additional file 3: Figure S1). These results suggested a mechanism, where AR might be a necessary medium in FOXA1-enhanced Notch pathway activation in AN3CA cells.

### FOXA1 promotes proliferation of human EC cells

**Figure 5:**
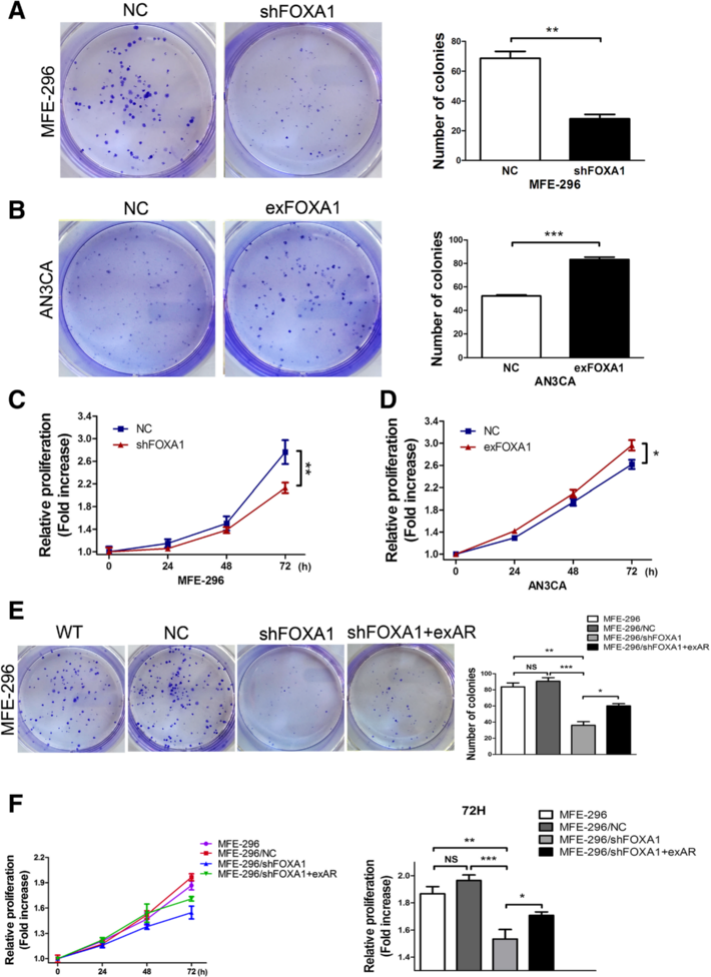
**FOXA1 promotes proliferation of human EC cells by affecting AR-mediated transcription. A**: Proliferation in MFE-296 cells transfected with NC or shFOXA1 was assessed by the colony-forming assay (Left) and further quantified in the number of colonies of triplicate experiments (Right). **B**: Proliferation in AN3CA cells transfected with NC or exFOXA1 was assessed by the colony-forming assay (Left) and further quantified in the number of colonies of triplicate experiments (Right). **C**: Assessment of proliferation by the MTT assay in MFE-296 cells transfected with NC or shFOXA1. **D**: Assessment of proliferation by the MTT assay in AN3CA cells transfected with NC or exFOXA1. **E**: Left: Colony-formation assay of untransfected MFE-296 cells (WT) and MFE-296 cells transfected with NC, shFOXA1, or shFOXA1 and exAR. Right: Graphical representation of the fold change in the number of colonies in untransfected MFE-296 cells (MFE-296) and MFE-296 cells transfected with NC (MFE-296/NC), shFOXA1 (MFE-296/shFOXA1), or shFOXA1 and exAR (MFE-296/shFOXA1 + exAR). **F**: Proliferation of MFE-296, MFE-296/NC, MFE-296/shFOXA1, or MFE-296/shFOXA1 + exAR cells was assessed by MTT assay. The right panel reiterates the data in the left panel at 72 h. *p < 0.05, **p < 0.01, ***p < 0.001 and NS p > 0.05.

To examine the role of FOXA1 in EC cell proliferation, we assessed the effect of FOXA1 in colony-forming and MTT assays. In the colony-forming assay, MFE-296 cell transfected with shFOXA1 showed significantly decreased colony-forming ability when compared with MFE-296 cells transfected with NC (Figure 5A). Moreover, upregualtion of FOXA1 in AN3CA cells showed increased colony-forming ability compared with NC cells (Figure 5B). In the MTT assay, downregulation of FOXA1 in MFE-296 cells resulted in poor cell viability (Figure 5C), and upregulation of FOXA1 in AN3CA cells caused increased cell viability (Figure 5D). These data indicated that FOXA1 promoted cell proliferation.

### AR is required for FOXA1-enhanced proliferation of EC cells

To directly address whether the effects of FOXA1 in promoting EC cell proliferation can be attributed to its activation of AR, a rescue experiment in MFE-296 cells was performed. In the colony-forming assay, cotransfection with exAR rescued the decreased rate of cell growth caused by FOXA1 downregulation in shFOXA1 cells (Figure 5E). The MTT assay also showed that cotransfection with exAR rescued the inhibition of cell viability caused by FOXA1 downregulation in shFOXA1 cells (Figure 5F). The similarity of results from the colony-forming and MTT assays suggested that the effects of FOXA1 in mediating cell proliferation of EC cells were mediated through AR.

### AR is not required for FOXA1-enhanced migration and invasion of EC cells

**Figure 6:**
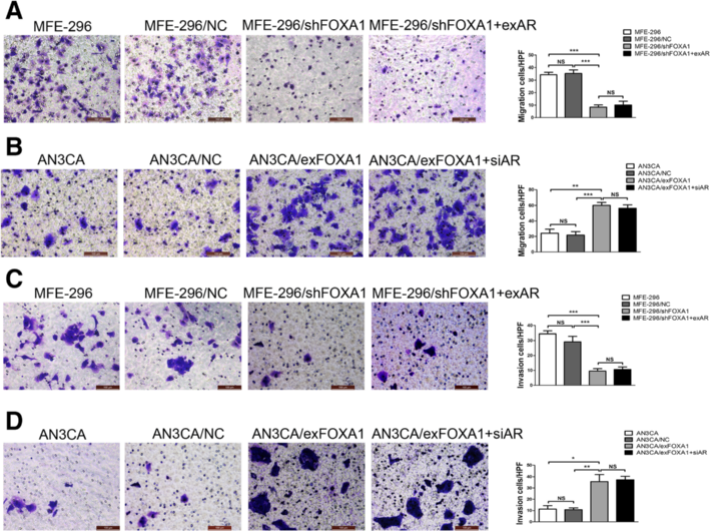
**FOXA1 induces migration and invasion in EC cells. A**: Cell migration of MFE-296, MFE-296/NC, MFE-296/shFOXA1 and MFE-296/shFOXA1 + exAR cells was assessed by the transwell migration analysis (Left). The mean ± SD number of migrated cells of three independent experiments was showed in the right panel. The abbreviation “HPF” on the *y* axis means one high power field. **B**: Cell migration of AN3CA, AN3CA/NC, AN3CA/exFOXA1 and AN3CA/exFOXA1 + siAR cells were subjected to transwell migration analysis (Left). The mean ± SD number of migrated cells of three independent experiments was showed in the right panel. **C**: Cell invasion of MFE-296, MFE-296/NC, MFE-296/shFOXA1 and MFE-296/shFOXA1 + exAR cells was assessed by the transwell invasion analysis (Left). The mean ± SD number of invased cells of three independent experiments was shown in the right panel. **D**: Cell invasion of AN3CA, AN3CA/NC, AN3CA/exFOXA1 and AN3CA/exFOXA1 + siAR cells by three independent experiments were subjected to transwell invasion analysis (Left). The mean ± SD number of invased cells of three independent experiments was showed in the right panel. (Magnification, 200×). *p < 0.05, **p < 0.01, ***p < 0.001, and NS p > 0.05.

Our immunohistochemistry results revealed that patients with myometrial invasion displayed higher FOXA1 expression. With this observation in mind, we hypothesized that functional expression of FOXA1 might induce tumor metastasis in EC. To explore the role of FOXA1 in the regulation of metastatic function and to determine whether AR is involved in FOXA1-mediated regulation of metastatic function, we examined the migration and invasion ability of MFE-296/shFOXA1 and AN3CA/exFOXA1 cells after exAR or siAR cotransfection using transwell migration and invasion assays. MFE-296/shFOXA1 cells displayed a decreased rate of migration compared to MFE-296/NC cells (Figure 6A). However, cotransfection of MFE-296/shFOXA1 cells with exAR (MFE-296/shFOXA1 + exAR) did not rescue the migration to the levels observed in MFE-296/NC or untransfected cells (MFE-296) (Figure 6A). Furthermore, AN3CA/exFOXA1 cells exhibited a high migration rate as compared with AN3CA/NC cells, but cotransfection with siAR (AN3CA/exFOXA1 + siAR) did not significantly attenuate the migration rate (Figure 6B).

Consistent with these findings, the invasion rate was significantly reduced in MFE-296/shFOXA1 cells, but the reduction was not reversed upon transfection with exAR (Figure 6C). Likewise, the invasion rate was enhanced in AN3CA/exFOXA1 cells, but this enhancement was not attenuated upon transfection with siAR (Figure 6D). These results demonstrated a functional role for FOXA1 in mediating migration and invasion in EC cells and suggested a mechanism (distinct from that for EC cell proliferation) by which AR might not contribute to FOXA1-mediated metastasis of EC.

### Oncogenic role of FOXA1 in a tumor xenograft model

**Figure 7:**
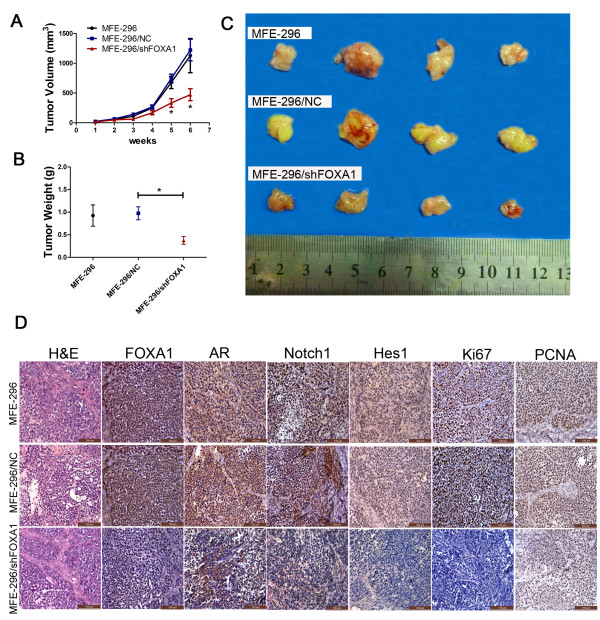
**Tumorigenicity assay in nude mice. A**: The growth rates of tumors formed from untransfected MFE-296 cells (MFE-296) and MFE-296 cells transfected with NC (MFE-296/NC) or shFOXA1 (MFE-296/shFOXA1). After injection, tumor volumes were calculated every seven days. **B** and **C**: Six weeks after injection of MFE-296, MFE-296-NC, and MFE-296-shFOXA1 cells, tumors were removed, and the tumor weights and volumes were determined. Arithmetic means and SD are shown. **D**: Staining with hematoxylin and eosin (H&E) or immunohistochemical staining for FOXA1, AR, Notch1, Hes1, Ki67, and PCNA in mouse tumor tissues (immunohistochemical staining, 200×). *p < 0.05 compared with the NC group.

Tumors generated by subcutaneous implantation of MFE-296 cells were used to evaluate the effect of FOXA1 on proliferation in a mouse tumor xenograft model. We measured tumor volumes in xenografted mice over a 6-week period following injection of untransfected MFE-296 (MFE-296), stably transfected with shFOXA1 (MFE-296/shFOXA1) or NC (MFE-296/NC). These measurements indicated that tumors in the MFE-296/shFOXA1 group grew significantly slower than those in the MFE-296/NC group and the MFE-296 group (Figure 7A). Six weeks after injection, tumors were removed from the mice (Figure 7C). The final mean weight and volume of tumors in the MFE-296/shFOXA1 group were significantly lower than those in the MFE-296/NC group (p < 0.05, Figure 7B and 7C). Tumor tissues were then embedded in paraffin, stained with hematoxylin and eosin (H&E), and immunohistochemically stained with antibodies against FOXA1, AR, Notch1, Hes1, Ki67, or PCNA. Lower FOXA1 expression in the MFE-296/shFOXA1 group also led to reduced staining for AR, indicating that FOXA1 also affected AR expression in vivo, in accordance with the results in vitro. As expected, the MFE-296/shFOXA1 group had significantly lower levels of Notch1 and Hes1 (Figure 7D), thus verifying the role of FOXA1 as a positive regulator of the Notch pathway in vivo. Furthermore, to determine the proliferative ability of MFE-296 cells, we performed immunohistochemical staining of Ki67 and PCNA, which are expressed as proliferation indices. The observed lower expression of Ki67 and PCNA in the MFE-296/shFOXA1 group was consistent with the smaller tumor volumes in the mouse tumor xenograft model (Figure 7D).

## Discussion

Over the past decade, FOXA1 expression has been examined in several human cancers, and oncogenic and tumor-suppressive roles have been proposed for FOXA1 depending on the cancer type and, in some cases, the subtype. In acute myelocytic leukemia, esophageal squamous cell carcinomas, lung adenocarcinomas, thyroid carcinoma, prostate cancer, and AR-positive molecular apocrine breast cancer [12,23-25], FOXA1 acts as an oncogene. However, in hepatocellular carcinoma, pancreatic, and estrogen receptor (ER)-positive breast cancer, FOXA1 has been reported to have a tumor-suppressive function [26-28]. On one hand, FOXA1 acts as a tumor oncogene. In oesophageal squamous cell carcinoma, FOXA1 expression is correlated with lymph node metastases in immunohistochemical specimens and FOXA1 expression inhibition decreases cellular invasion and migration [29]. Also, FOXA1 is over-expressed in aggressive thyroid cancers (ATC) and involved in cell cycle progression via down-regulation of p27^Kip1^ in an ATC cell line [30]. On the other hand, FOXA1 has been reported to act as a tumor suppressor. It has been reported that FOXA1 positively regulates miRNA-122, which is correlated with favourable prognosis in human hepatocellular carcinoma [26]. In addition, FOXA1 acts as an important antagonist of the epithelial-to-mesenchymal transition (EMT) in pancreatic ductal adenocarcinoma through its positive regulation of E-cadherin and maintenance of the epithelial phenotype [27]. It is critical to note that the role of FOXA1, as a tumor oncogene or a tumor suppressor gene, has been reported to vary in prostate and breast cancers depending on multiple cancer subtypes and states of hormone dependence or independence [11,12,28].

A previous study has addressed the expression and function of FOXA1 in EC; immunohistochemical analysis by Abe et al. indicated that FOXA1 was negatively associated with lymph node status in EC immunohistochemical specimens in Japanese, and FOXA1 repressed proliferation and migration in one type of EC cells (Ishikawa) [31]. However, our study found that the FOXA1 level in ECs was significantly higher than that in atypical hyperplasia and normal tissues (p < 0.05) in immunohistochemical specimens and that FOXA1 promoted tumor cell proliferation in EC, which differs from the previous results. The difference might be attributed to the immunohistochemical samples in different countries used. Alternatively, the cancer subtype may affect the results: the function of FOXA1 as a tumor suppressor in the Abe et al. study was investigated in the Ishikawa cell line, which is ER-positive [32], whereas we used MFE-296 (high levels of FOXA1 and AR) and AN3CA (low levels of FOXA1 and AR), which are both ER-negative cell lines [33,34]. This idea consists with breast cancer studies that have shown that FOXA1 functions as a tumor suppressor in ER-positive breast cancer cells (MCF-7) [28] but as a tumor activator in ER-negative breast cancer cells (MDA-MB-453) [12]. Furthermore, this idea of the effects of forkhead family members depending on ER expression is also consistent with the study that have shown the Forkhead box class o 3a transcription factor (FoxO3a) has inhibitory effects on motility and invasiveness of ER-positive breast cancer cells but inducing effects on motility and invasiveness of ER-negative breast cancer cells [35]. More comprehensive studies covering several EC cell lines in different cancer subtypes will be necessary to define the role of FOXA1 in EC development.

Most researches on hormone receptors in EC have focused on ER and progesterone receptor (PR). However, the expression of AR in the human normal endometrium and its disorders is not well understood. Though higher serum androgen levels have been certified to exist in the utero-ovarian vein blood samples from women with EC [36], the details of AR expression and its actions in EC are a topic of dispute. Longer CAG repeats in AR promote carcinogenesis of uterine endometrial cells [37]. Androgens and AR may be involved in endometrial cell proliferation by regulating the expression of insulin growth factor I (IGF-I) in the uterus [38]. Our results suggest that AR expression is significantly higher in EC than in normal endometrium and that AR activated by FOXA1 might promote the Notch pathway, which may be another mechanism involving AR in EC.

Most FOXA1 studies have focused on its role as a pioneer factor that binds to DNA packaged in chromatin and opens the chromatin for binding of additional transcription factors including AR [39,40]. According to our results from qRT-PCR and western blotting, FOXA1 regulates AR target genes by up-regulation of AR expression. Interestingly, our co-immunoprecipitation results (Figure 4A and 4B) showed that FOXA1 interacted with AR at the protein level. Apart from that, our ChIP-PCR results suggested that FOXA1 and AR were directly bound to the same regions upstream of MYC (Figure 4C and 4D). Based on the above results, we suggest that FOXA1 may also directly regulate AR target genes (at least MYC) by binding to AR in EC. Our results regarding an interaction between AR and FOXA1 may be related to the finding that the AR and FOXA1 binding sites are adjacent on multiple promoters of AR target genes in prostatic cells [9,41]. Thus, FOXA1 may regulate the AR target genes through at least two means: AR over-expression or physical interaction with AR in order to induce easy AR accessibility to binding to its target genes. MYC is an immediate early response gene downstream from AR pathway and is tightly regulated through AR cis-regulatory elements identified within its proximal promoters and distal enhancer regions [19], which is consistent with our ChIP-PCR results (Figure 4C and 4D). Interestingly, we showed that FOXA1 and AR more evidently bound to the MYC enhancer regions as compared to MYC promoter regions. These results could be attributed to other co-regulators involved in this binding process. Since TCF7L2, a protein mediating DNA looping for long-distance interactions of distal enhancers and proximal promoters, physically interacts with FOXA1 and AR and mediates the transcription of MYC in breast cancer [19], future investigation will be needed to clarify which co-regulators are involved in FOXA1/AR binding to the enhancer regions upstream of MYC in EC cells.

Although the underlying mechanisms governing the FOXA1-AR correlation in tumor progression are not fully understood, a pathway analysis showed that 187 FOXA1/AR dual target genes were involved in the cellular growth/proliferation pathway in liver cancer [22]. The Notch pathway is implicated in the development of various cancers, and the Notch pathway blockade appears to affect cell proliferation in multiple types of cancers. Notch pathway inhibition in breast cancer cells induces cell cycle arrest and apoptosis [42]. Similarly, downregulation of Notch1 contributes to cell growth inhibition in pancreatic cancer [43]. Our results suggest that downregulation of AR attenuated FOXA1-induced upregulation of the Notch pathway in EC cells. These findings indicate that FOXA1 might promote AR-mediated transcription and ultimately activate the Notch pathway. Here, we describe, for the first time, the association between FOXA1 expression and the Notch pathway in cancer.

The specific mechanism of cell proliferation in EC reported so far has been limited, although several classical transcription factors related to proliferation have been identified, including cyclin D1, p53, IGFBP-1, PTEN, and p27^Kip1^[44-48]. In this study, we suggest that FOXA1 promotes cell proliferation in EC by interaction with AR, possibly via the Notch pathway, which may be a newly identified regulatory mechanism of cell proliferation in EC.

We further investigated the effects of FOXA1 and AR on migration and invasion of EC cells, and found that neutralization of AR activity did not inhibit FOXA1-enhanced cancer cell migration or invasion. These observations indicate that the promoting effect of FOXA1 on migration and invasion is not dependent on AR. Our findings in migration and invasion assays are consistent with our findings in immunohistochemical staining, which showed that higher expression of FOXA1 but not AR is found in tumors that displayed a greater depth of myometrial invasion. These results suggest that AR is not the only downstream target of FOXA1 in EC. Future studies will be necessary to define which transcription factors or pathways are involved in FOXA1-enhanced cell migration and invasion in EC.

The traditional endocrine treatment (mainly targeting ER and PR) is ineffective in most ER-negative and PR-negative ECs, and even in some ER-positive and PR-positive ECs [49]. In our investigation, 9 of the15 ER-negative EC cases (60.0%) and 41 of the 61 ER-positive EC cases (67.2%) were AR positive, and the majority of ECs were also FOXA1 positive (Table 1). Thus, AR and FOXA1 might be alternative targets in ECs insensitive to traditional endocrine treatment or could be targets for adjuvant treatment following surgery and traditional endocrine treatment. There has been speculation about the use of anti-androgens for the treatment of ECs [50]; this hypothesis warrants clinical investigation in light of our findings.

## Conclusions

In summary, our results suggest a new mechanism for the development of EC, in which FOXA1 promotes tumor cell proliferation through AR and activates the Notch pathway by influencing AR expression. The newly identified FOXA1-AR interaction will help further elucidate the molecular mechanisms underlying EC progression and suggests that FOXA1 and AR are potential targets for EC treatment.

## Competing interests

The authors declare that they have no competing interests.

## Authors’ contributions

MQ, WB, and JW carried out the design of the experiments, performed most of experiments, and drafted the manuscript. TY and XH participated in the molecular biology experiments and statistical analysis. YL made the figures. XW was involved in financial support, the design of the experiments, data analysis, and final approval of the manuscript. All authors read and approved the final manuscript.

## Pre-publication history

The pre-publication history for this paper can be accessed here:

http://www.biomedcentral.com/1471-2407/14/78/prepub

## Supplementary Material

Additional file 1: Table S1 Statistical difference between FOXA1 expression in normal endometrium and endometrial cancer. Click here for file

Additional file 2: Table S2 Statistical difference between AR expression in normal endometrium and endometrial cancer. Click here for file

Additional file 3: Figure S1 AR is a necessary medium in FOXA1-enhanced Notch pathway activation. Protein levels of AR, FOXA1, Notch1, and Hes1 in untransfected AN3CA cells (AN3CA) and AN3CA cells transfected with NC (AN3CA/NC), exAR (AN3CA/exAR), or exAR and siFOXA1 (AN3CA/exAR + siFOXA1) were measured by western blotting (Right), and were further quantified by densitometry of triplicate experiments (Left). β-actin was used as a loading control. *p < 0.05, ** p < 0.01, ***p < 0.001, and NS p > 0.05. Click here for file
