# Supplementary figures and images for: FOXA1 promotes tumor cell proliferation through AR involving the Notch pathway in endometrial cancer
Source: BMC Cancer. 2014 Feb 11;14:78. doi: 10.1186/1471-2407-14-78 (PMC3926330; doi:10.1186/1471-2407-14-78)

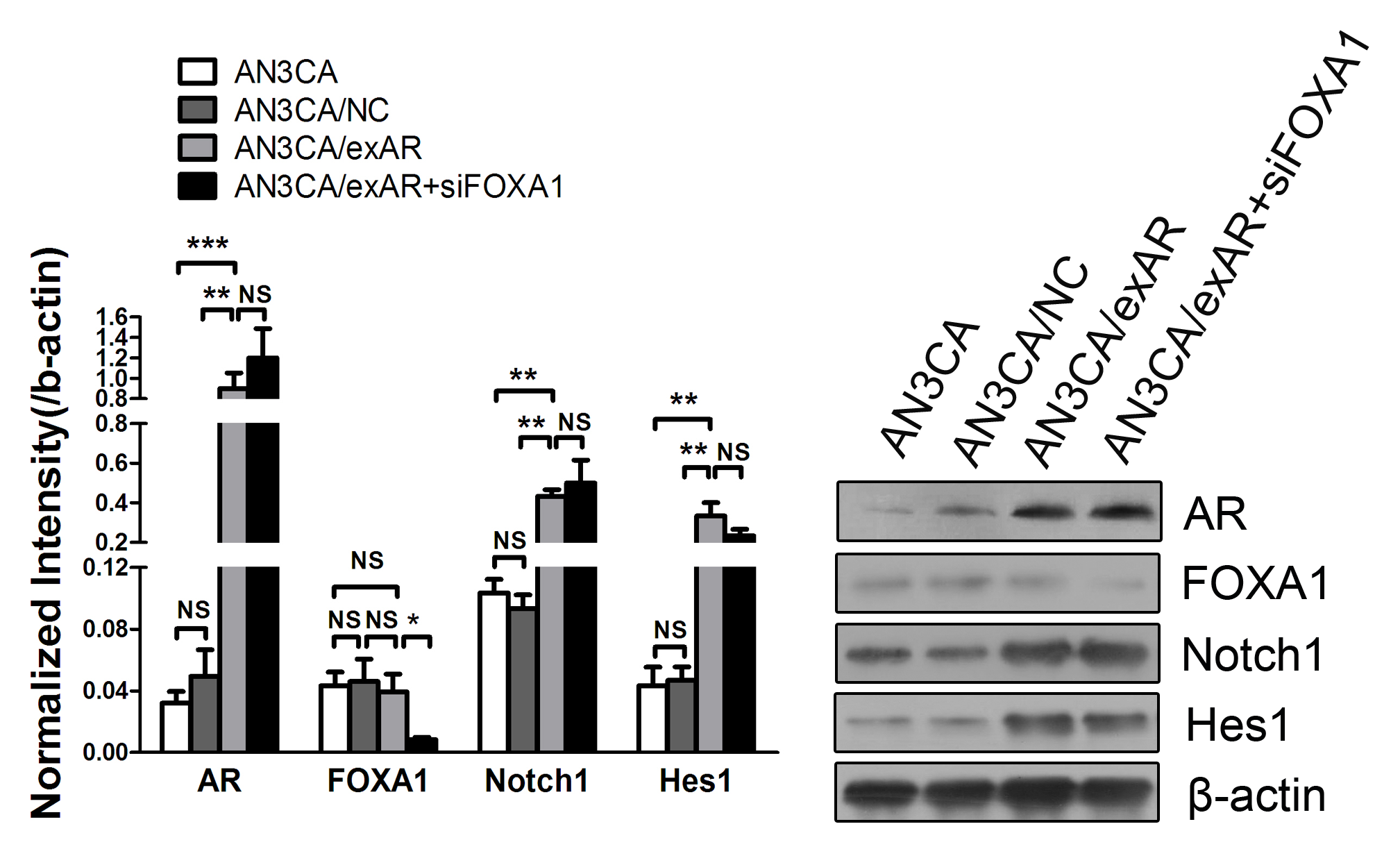

Supplement: Additional file 3: Figure S1 — AR is a necessary medium in FOXA1-enhanced Notch pathway activation. Protein levels of AR, FOXA1, Notch1, and Hes1 in untransfected AN3CA cells (AN3CA) and AN3CA cells transfected with NC (AN3CA/NC), exAR (AN3CA/exAR), or exAR and siFOXA1 (AN3CA/exAR + siFOXA1) were measured by western blotting (Right), and were further quantified by densitometry of triplicate experiments (Left). β-actin was used as a loading control. *p < 0.05, ** p < 0.01, ***p < 0.001, and NS p > 0.05. [file 1471-2407-14-78-S3.tiff]
